# Transformation of bimetallic Ag–Cu thin films into plasmonically active composite nanostructures

**DOI:** 10.1038/s41598-023-37343-2

**Published:** 2023-06-21

**Authors:** Marcin Łapiński, Robert Kozioł, Wojciech Skubida, Piotr Winiarz, Rowa Mahjoub Yahia Elhassan, Wojciech Sadowski, Barbara Kościelska

**Affiliations:** 1 Institute of Nanotechnology and Materials Engineering, Advanced Materials Center, Gdansk University of Technology, ul. G. Narutowicza 11/12, 80-233 Gdańsk, Poland; 2 Department of Hydrogen Energy, Faculty of Energy and Fuels, AGH University of Science and Technology, al. A. Mickiewicza 30, 30-059 Kraków, Poland; 3 Department of Physical and Chemical Science, University of L’Aquila, Via Vetoio 10, 67100 L’Aquila, Italy

**Keywords:** Nanophotonics and plasmonics, Nanoparticles, Two-dimensional materials

## Abstract

Formation of plasmonically active silver, copper and composite silver-copper nanostructures were studied in this paper. Metallic nanostructures were fabricated by thermal disintegration, so called dewetting, of the thin films in an argon atmosphere. The formation process of the nanostructures was in-situ observed by a novel method, based on resistance measurements. The influence of the material and thickness of the initial thin film on temperature of their disintegration was investigated. Electrical measurements were validated by scanning electron microscopy observations, while metallic the behavior of nanostructures was studied by XPS method. The formation of silver-copper nanocomposite structures was confirmed by UV–vis spectroscopy. Plasmon resonance with two characteristic peaks for nanocomposite structures was observed.

## Introduction

Noble metals nanostructures are widely used in plasmonic applications. However, their fixed optical properties, such as the position of plasmon resonance or the extinction coefficient of monometallic nanostructures, currently limit their use in photonic devices. To achieve plasmonic structures with tuneable optical response, nanoalloyed and nanocomposited structures can be considered^1–4^. For metallic nanostructures, the extinction coefficient, as well as the plasmon resonance wavelength, depends on the dielectric properties of the environment around nanostructures, the dielectric function of material of nanostructures and their shape and size. For the spherical nanostructures, plasmonic behaviour can be described by the dipolar approximation of the Mie law, given by Garcia^5^:

1 $$\upsigma =\frac{24{\pi }^{2}{R}^{3}{\varepsilon }_{e}^{3/2}}{\lambda }\frac{{i\varepsilon }_{2}}{{\left({\varepsilon }_{1}+2{\varepsilon }_{e}\right)}^{2}+{i\varepsilon }_{2}^{2}},$$

where σ is the extinction (the sum of optical absorption and scattering), R is the radius of nanostructures, ε_e_ is the dielectric constant of the environment, ε_1_ + iε_2_ is the complex dielectric function of metal and λ is the resonance wavelength^5^.

**Figure 1:**
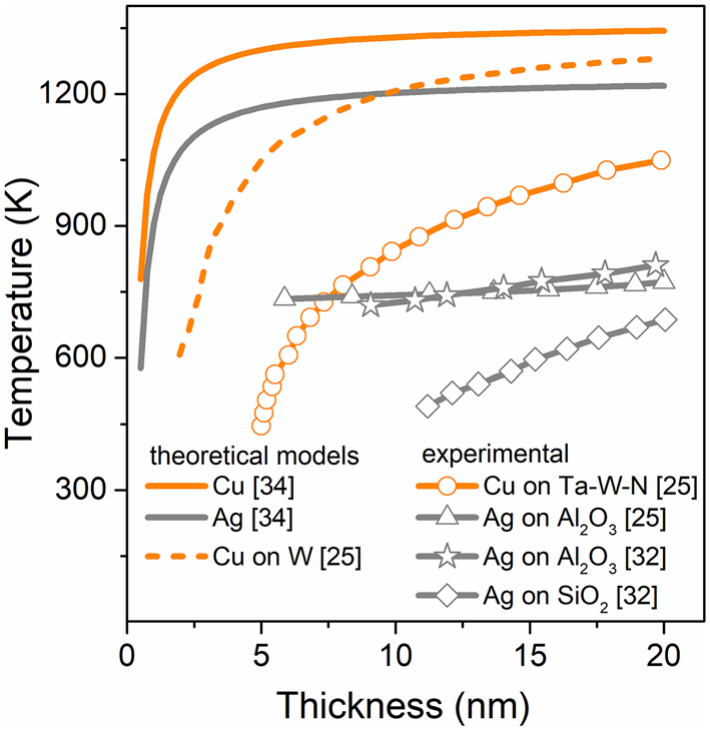
Melting temperature of copper and silver thin films in a function of their thickness. Data of theoretical calculations and experimental results from Refs.^25,32,34^.

Ignoring the effects of environment (ε_e_) and size (R), the optical properties of metallic nanostructures are mainly determined by its free electrons and bound electrons. In alloyed nanostructures atoms create a new structure due to the mixing of free electrons and energy level hybridization^6^. It results in the appearance of new optical properties. Changes in electronic structure influence the variation of dielectric function. Possibility to modulate the dielectric function (ε = ε_1_ + iε_2_) in alloyed nanostructures would allow to control the plasmonic response of nanostructures. It opens a new possibilities for fabricating a optoelectronic devices with unique properties, like for example, metamaterials for tuneable absorbers and optical filers, high-performance detectors or high efficiency solar cells^3,7–9^. Another fascinating area of applications for nanoalloyed and nanocomposite structures could be photocatalysis^10–12^. Also in plasmonic applications, nanoalloys and nanocomposites may spark an interests. While the alloyed nanostructures have a different dielectric function from the metals used to create them, nanocomposites conserve the dielectic function of base materials^13^. Nanocomposites could be considered as a quite small grains creating bigger nanostructure or as a core–shell structures. The plasmon response of bimetallic nanocomposites and its dependence on the grain or the unit size are still a subject of intensive scientific research^13–16^. Among various techniques, the thermal evolution of bimetallic thin films seems to be one of the most promising method for nanocomposites production. It has been recently studied and is well known, that thermal annealing results in thin-film transformation into droplets or islands^17–20^. The transformation mechanism is driven by thermally accelerated diffusion that leads to the minimalization of surface energy in the system^21–24^. However, there is no clearly explained, step by step, mechanism for this process, as well as no common terminology for it. This phenomenon can be named as an agglomeration^25,26^, thin film disintegration^27^, rupture^28^, self-organisation^29^, dewetting^7,30,31^ or melting^32^. There is no even consensus on whether thin film transformation into droplets occurs in the liquid or solid phase^17,32,33^. In Fig. 1 theoretically calculated and experimentally measured melting temperatures of the thin film in a function of thickness for Ag and Cu are compared. To determine the melting temperature, Qi proposed a model based on size-dependent cohesive energy^34^, while Gromov and Kitsyuk presented both, theoretical and experimental results^25,32^. One can see a strong correlation between layer thickness and melting point. In particular, it may be note that the nature of the curve is similar^35,36^. Theoretically calculated values are higher, than experimental, probably due to not taking all factors into account in models. Additionally on experimental results influence of substrate material is visible, due to various surface tensions.

Based on our previous experiments with gold, silver and bimetallic Au–Ag nanostructures, we can explain the formation of metallic islands from thin films as a nucleation of voids in the film’s surface. Holes appear in the grain boundaries, most likely at the triple junctions of boundaries. It can be explained as a surface melting or premelting in a region with a lower film thickness^37–39^. Voids expand and the material diffuse and create a raised ridge. These ridges transform into isolated islands. As a result of the melting of the nanoislands at temperatures well below melting point of the bulk body, nanostructures evolved into spherical droplets^2,7,31,40,41^. Moreover, the process can be more complicated for multimetallic films, when eutectics must be taken into account. It could play a key role especially in nanodimensional systems, when the eutectic temperature drastically decreases, as well as the eutectic composition changes, with size reduction^42–45^.

In this work we present the results of our study of the transformation of silver, copper and bimetallic, silver-copper thin films into nanoislands. Due to the high segregation factor, in contrast to e.g. gold-silver systems, copper and silver forms not nanoalloys, but nanocomposites^46–48^. We proposed a novel method, based on electrical measurements, for in situ observations of disintegration of the thin films. It could be described as the breaking of electrical conductivity of a metallic film. The method was validated by scanning electron microscopy, while the plasmon resonance of manufactured nanostructures, was controlled by UV–Vis optical spectroscopy.

### Experimental

Thin films were deposited on a both type substrates: silicon (111) for SEM and XPS observations and Corning 1737 glass for electrical and optical measurements. Substrates were cleaned with acetylacetone and then rinsed in isopropyl alcohol. For electrical measurements, four strip gold electrodes, with a width of 0.8 mm and a thickness of 200 nm were evaporated on glass substrates. The distance between electrodes was 1 mm. Thin silver, copper and bimetallic Ag–Cu films with a various thickness were sputtered with the use of a table-top dc magnetron sputtering coater (EM SCD 500, Leica) in the pure Ar plasma state (Argon, Air products 99.99%), at room temperature. The Cu and Ag targets had 99.99% purity. The sputtering process was carried out with an incident power in the range of 12–15 W, which provided a film deposition rate of approximately 0.1 nm per second for Cu, and ca. 0.2 nm per second for Ag. The magnetron coater was equipped with a quartz crystal microbalance, which provided in-situ film thickness measurements. For experiments, pure silver and copper films with a thickness in a range of 2–20 nm were selected. Additionally bilayer structures with thickness ratio of 50% silver and 50% copper (Ag–Cu samples) were produced for tests. As a bottom layer copper was deposited and silver film was sputtered as a top layer. In order to form plasmonic nanostructures, as prepared films of varying thicknesses were subsequently put to the hot furnace. Samples were annealed at various temperatures for 15 min in an ambient argon gas atmosphere (Argon, Air products 99.99%).

In order to analyse the formation of nanostructures from thin films, electrical measurements were performed. Electrical resistance of the mono- and bilayers was measured by the conventional four-point method by the Keysight 3490A multimeter. In place of evaporated electrodes, wires were connected to films with silver paste. The measurements were performed at a temperature range of up to 900 K, controlled with a thermocouple type K placed in direct vicinity of the sample, with a heating rate of 5 K per minute in a pure argon atmosphere. Data was collected with a rate of 1 s.

To observe the morphology and evolution of the thin films, SEM images were collected by FEI Quanta FEG 250 scanning electron microscope. The microscope was operated at 10 kV. Additionally the energy dispersive X-ray spectrometer (EDS) for elemental analysis of nanoislands was used.

The chemical composition of samples was confirmed by the X-ray photoelectron spectroscopy (XPS) method. Measurements were carried out with an X-ray photoelectron hemispherical spectrometer (Argus Omicron NanoTechnology) with a Mg-Kα source of X-ray and anode operated at 15 keV, 300W. XPS measurements were conducted under an ultra-high vacuum at room temperature, with pressure below 1.1 × 10–8 mbar. Results were calibrated to C1s line and analysed by Casa XPS software package.

Plasmonic properties of UV–Vis spectra were recorded with a Thermo Fisher Scientific Evolution 220 double beam spectrophotometer in the transmittance mode in the range of 200 nm–1000 nm.

## Results

**Figure 2:**
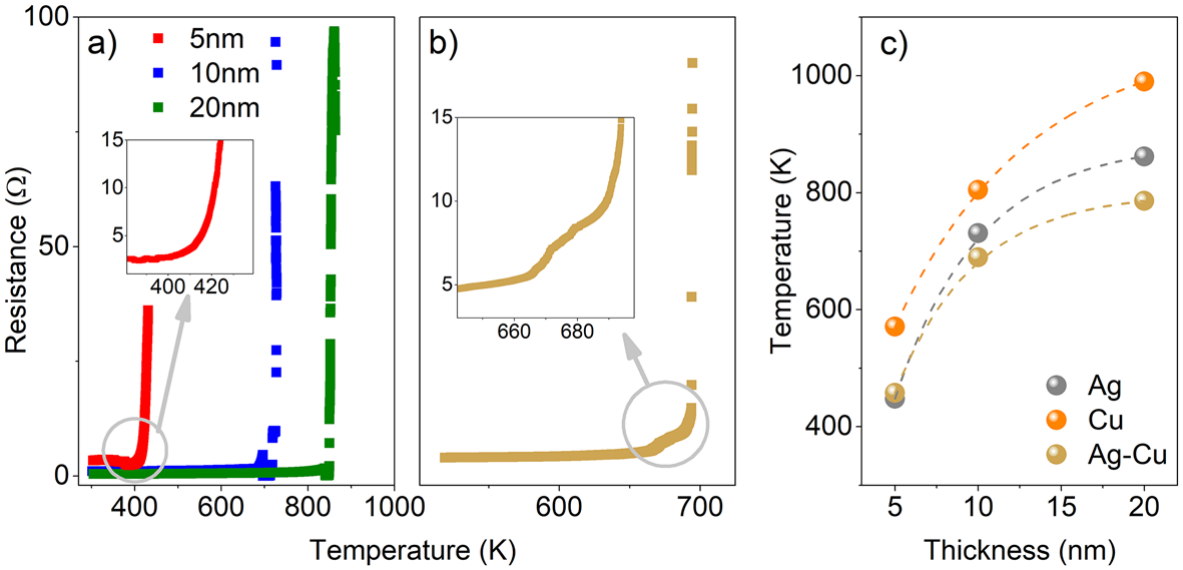
(**a**) Temperature dependence of resistance for Ag thin films with thickness of 5 nm, 10 nm and 20 nm; (**b**) Temperature dependence of resistance for Ag–Cu thin film with a thickness of 10 nm; (**c**) Thickness dependence of the breaking of the electrical continuity for Ag, Cu and Ag–Cu thin films.

Disintegration of the metallic or bimetallic thin films was observed by a nonconventional approach—by measurements of the electrical resistance. Drastically increasing of resistance of the film during its heating is associated with breaking electrical continuity. That phenomenon was observed for example by Wójcik et al. during impedance spectroscopy measurements^49^. In Fig. 2a results of resistance measurements in a function of temperature for selected metallic samples are presented. One can see a sharp increase of resistance and a strong correlation between the temperature of breaking of electrical continuity and thickness of films. In Fig. 2b resistance characteristic for Ag–Cu bilayer, with a total thickness of 10 nm is presented. Overall characteristic is similar to recorded for monolayers (Fig. 2a). On the other hand, close to breaking temperature characteristics for monolayer and bilayers are slightly different (see enlargements in Fig. 2a and b). For pure silver film the resistance increases steadily, while for bilayer two regions with a various slope can be observed. It is evidence for breaking continuity not at the same time for Ag and Cu layer. It can be assumed that for a 10 nm bilayer film, as a first, silver layer disintegrate, at temperature ca. 20 K lower, than copper layer. The melting point of silver is lower than for copper, which could be the reason for the faster disintegration of Ag film. Additionally, a top layer is more sensitive to premelting. Results of electrical measurements are compared in Fig. 2c, where onset points of sharp resistance increasing are collected. The obtained characteristics are in line with the theory and experiments done by others (Fig. 1). Lower breaking temperature for bilayers could be explained by uneven disintegration of top and bottom layers which could be considered as a almost separate films.

**Figure 3:**
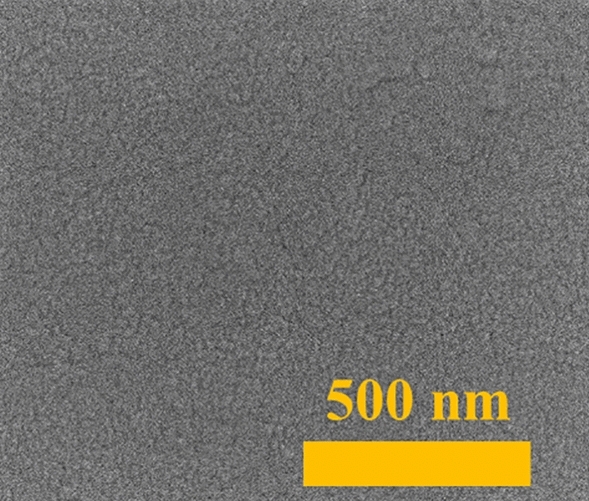
SEM image of the as-deposited silver film, with thickness of 8 nm.

**Figure 4:**
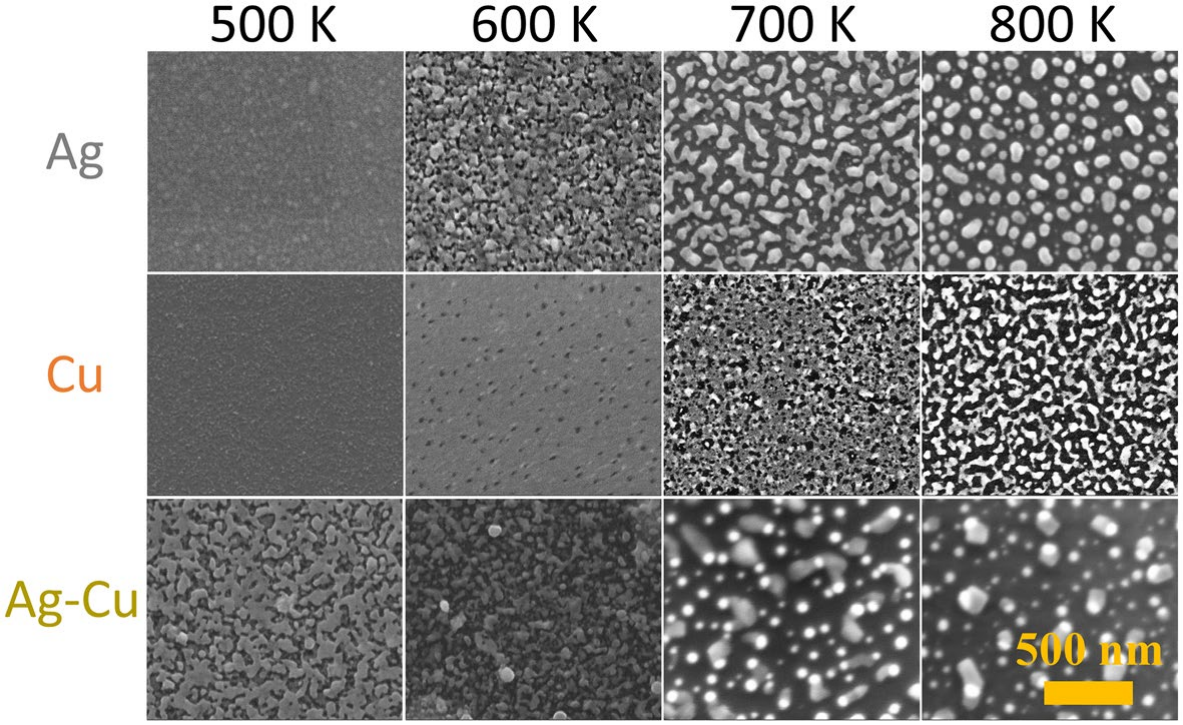
SEM images of the 8 nm Ag, Cu and Ag–Cu films at various temperature in a range of 500 K–800 K for 15 min.

**Figure 5:**
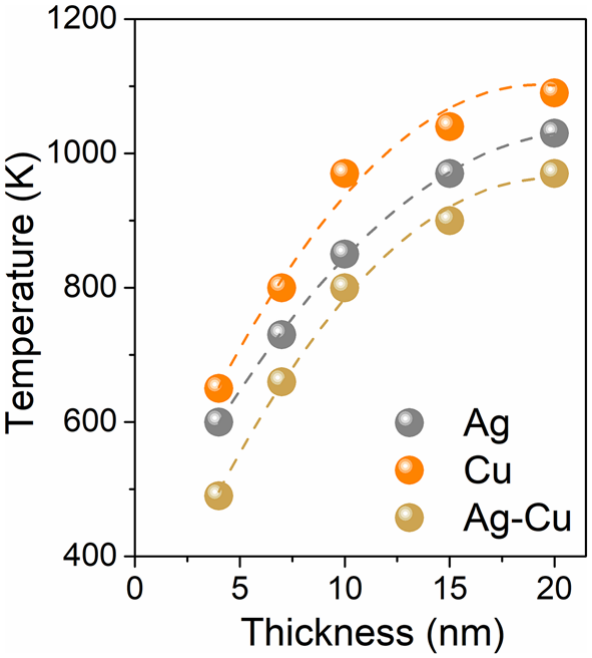
The influence of film thickness on the temperature of formation of Ag, Cu and Ag–Cu nanostructures. The temperature of nanostructure formation was determined on the basis of SEM images.

**Figure 6:**
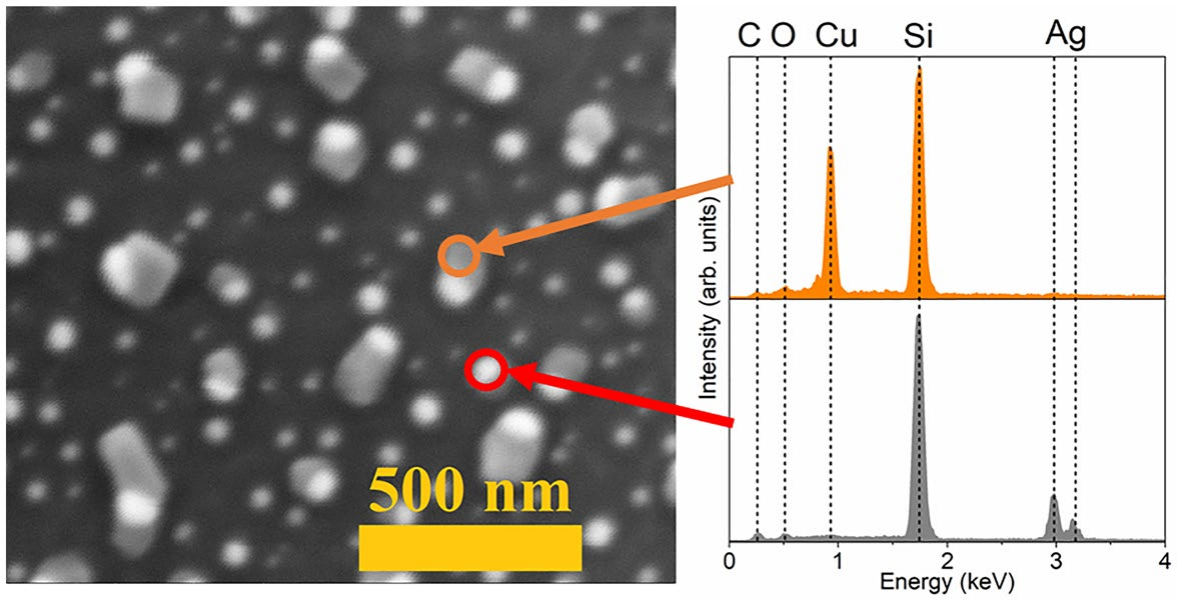
Results of the EDS analysis in a selected two regions of nanostructures obtained by annealing of the 8 nm Ag–Cu bilayer at 800 K.

In Fig. 3 an example SEM image of the as-deposited, 8 nm thick silver film is presented. As can be seen, a grain structure of the film is well visible, due to Stransky–Krastanov model of layer growth. That mixed, island-layer is characteristic for magnetron sputtered metallic films. Moreover, in Fig. 4 selected SEM images of nanostructures formed during annealing at different temperatures for 15 min of 8-nm silver and copper thin films and silver-copper bilayers are presented. One can see, that the process of forming nanostructures begins with ruptures in the layer, on the grain junctions. Then, voids are growing and initially irregular-shaped structures are formed. Finally, with the temperature increasing, separated metallic nanostructures are formed. The process looks similar for both metals, but the temperature at which the nanostructures form is different due to higher melting point of copper than silver. The influence of the initial metallic or bimetallic film thickness on the temperature of formation of nanostructures is shown in Fig. 5. The temperature of nanostructure formation was determined on the basis of SEM images. It was assumed that it is the temperature at which isolated islands are already completely formed, as is shown for example in Fig. 3 at 700 K for silver. The nature of characteristic presented in Fig. 5 is similar to characteristic prepared on the basis of electrical measurements, and presented in Fig. 2c. However temperatures determined from SEM observations seems to be higher, than from electrical measurements. This could be explained by difference in observation method. Isolated islands are formed at higher temperatures, than electrical continuity is broken. Additionally, a detailed elementary analysis were performed for nanostructures formed from Ag–Cu bilayers. On SEM images of the well-formed, bimetallic nanostructures with size from ca. 50 nm up to 100 nm, two regions can be distinguished. Bigger, elongated shape corresponding to copper and round spots consist silver, due to better substrate wetting by Cu than Ag. EDS point measurements confirmed a perfect separation of silver and copper in nanostructures (Fig. 6). Nanocomposites formation, due to high separation factor of Ag and Cu^46^ is a different result, than the creation of Au–Ag nanoalloys, as presented in our previous work^2^.

**Figure 7:**
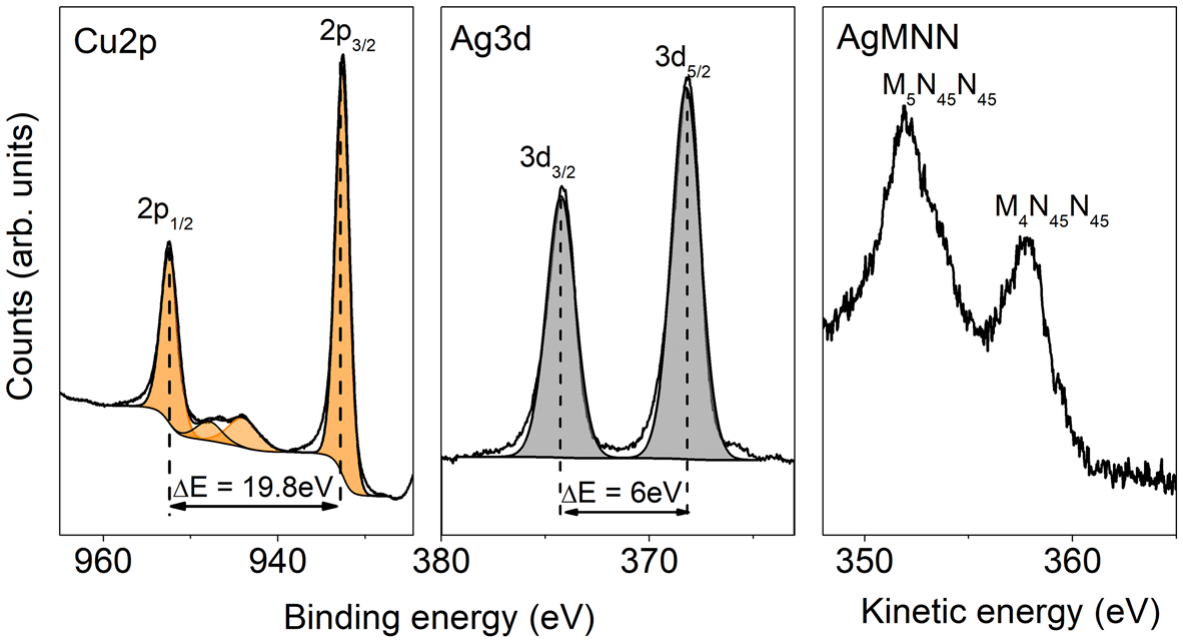
High resolution XPS spectra for Cu2p, Ag3d and AgMNN regions, recorded for nanostructures manufactured from Ag–Cu bilayers with total thickness of 8 nm, annealed at 800 K for 15 min.

XPS analysis conformed the metallic behaviour of manufactured structures. In Fig. 7 an example spectra for nanostructures formed in a result of thermal annealing at 800 K of bilayer with a total thickness of 8 nm (4 nm Cu and 4 nm Ag) are presented. The high resolution Cu2p spectrum consists of two peaks: 2p_3/2_ and 2p_1/2_ at 932.5 eV and 952.3 eV. The splitting energy of 19.8 eV and very weak satellite peaks between two main maxims is characteristic for metallic copper^50,51^. Silver 3d_5/2_ and 3d_3/2_ photoelectron peaks could be observed at 368.1 eV and 374.1 eV respectively. Separation distance of 6 eV between two components is characteristic for silver in metallic form^52,53^. Additionally, to confirm metallic state of silver, the Auger parameter (α) has been calculated according to the formula:

2 $$\alpha \, = \,{\text{BE}}\, + \,{\text{KE,}}$$

where BE is the binding energy of Ag3d_5/2_ peak and KE is the kinetic energy of Ag M_4_N_45_N_45_) peak^52,54,55^. The calculated parameter is 726 eV, what perfectly match to metallic silver^55,56^.

**Figure 8:**
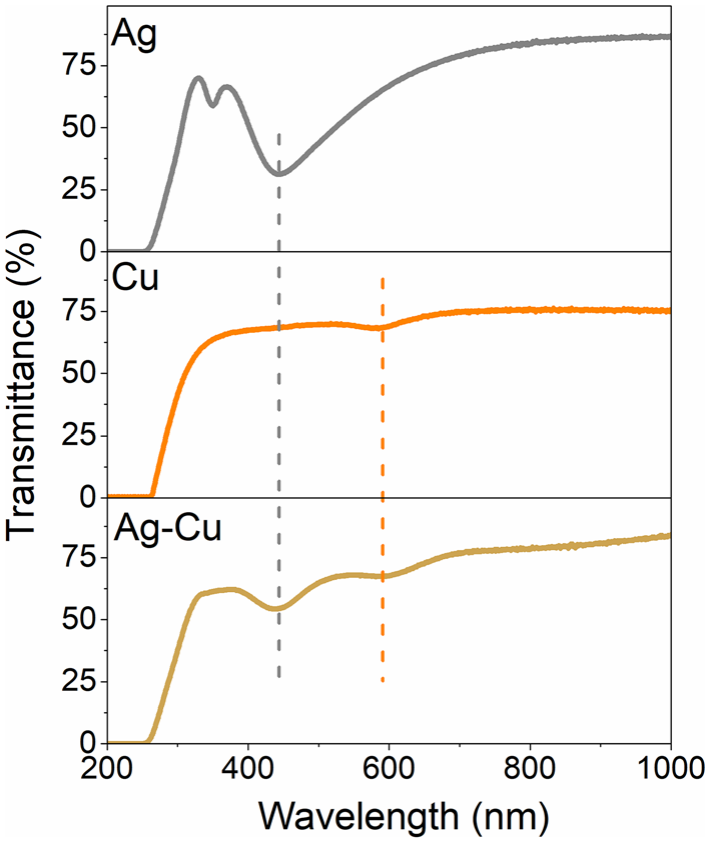
Transmittance spectra silver, copper and bimetallic silver coper nanostructures obtained during annealing of 8 nm thin films at 800 K for 15 min.

Plasmonic activity of manufactured nanostructures was measured by UV–vis spectroscopy method. As can be seen in Fig. 8 position of resonance for silver and copper based nanostructures is different, as well as, the intensity of resonance is various. It corresponds to results obtained by others^5,57–60^. Especially the multipolar resonance mode can be observed for silver nanostructures, due to its relatively large size^40^. Extinction coefficient of copper nanostructures is lower than for silver, what result in lower intensity of recorded resonance for Cu based nanomaterials^5^. The spectrum recorded for bimetallic nanostructures is an ideal composition of two spectra for Ag and Cu. Presence of two resonance minims, characteristic for silver and copper, indicates a nanocomposite, not a naoalloyed structure of islands^13,60^.

## Conclusions

Silver, copper and bimetallic silver-copper thin films were deposited by the magnetron sputtering method. The thermal instability of deposited layers was observed by a novel approach, based on electrical resistance measurements. It was compared the disintegration of monometallic film with that of bimetallic. It was found that bimetallic films disintegrate at a lower temperature, in comparison to silver or copper films with the same thickness. In the case of bilayers, the top (silver) and bottom (copper) layers dewetted at different times. As a result of thermal annealing of bimetallic layers, nanocomposite structures appeared. Formation was illustrated by the SEM images and metallic behaviour of nanostructures was affirmed by the XPS method. Additionally, the creation of composite nanostructures was confirmed by the optical method. The spectrum recorded for Ag–Cu nanostructures is an ideal composition of two spectra for Ag and Cu. Two resonance minima indicate the nanocomposite nature of nanostructures.

Our studies have shown that thermal disintegration of the metallic films can be observed by electrical method. Moreover, we have shown, that Ag–Cu thin films can be used to produce an composites structures with two plasmon resonance peaks.

## Data Availability

The datasets generated and/or analysed during the current study are available in the Transformation of bimetallic Ag–Cu thin films into plasmonically active composite nanostructures, https://doi.org/10.17605/OSF.IO/SY43F.
